# Spatial distribution of chickpea ascochyta blight (*Ascochyta rabiei*) and analyses of biophysical factors influencing disease epidemics in northwestern Ethiopia

**DOI:** 10.1371/journal.pone.0333002

**Published:** 2025-09-19

**Authors:** Addisu Mandefro, Getnet Yitayih, Girmay Aragaw

**Affiliations:** Department of Plant Sciences, Debre Tabor University, Debre Tabor, Ethiopia; Assam Agricultural University Faculty of Agriculture, INDIA

## Abstract

Chickpea (*Cice rarietinum* L.) is one of the important grain legume crops in Ethiopia, which serves as a source of both foreign exchange and food. However, the crop production and productivity are currently challenged by ascochyta blight disease caused by *Ascochyta rabiei* in the study areas.A total of 120 chickpea fields were assessed from five districts in two zonesduring the 2022 main cropping season to assess the distribution, prevalence, and intensity of ascochyta blight of chickpea and its association with agro-ecological factors in northwestern Ethiopia.The results confirmed a 100% prevalence of ascochytablight disease across districts. The highest disease incidence (46.32%) and severity (32.90%) were assessed from Fogera and GondarZuria districts, respectively. The associations between disease parameters and biophysical factors were performed using a binary logistic regression model.High incidence (>40%) and severity (>25%) were strongly associated with mixed cropping, Fogera and Gondar Zuria districts, ≤ two times land preparation, Vertisol soil type, desi chickpea type, and broadleaf weed type in the model. Lower disease incidence (≤ 40%) and severity (≤ 25%) had a strong association with sole cropping, more than two times land preparation, Nitisolsoil type, and growing of kabuli chickpea type. Thus, planting chickpea in sole cropping, more than two times land preparation, growing chickpea in Nitisol soil and use of kabuli chickpea typecould be used as management options to reduce the impact of the disease in northwestern Ethiopia and other similar ecological areas of the country.

## Introduction

Chickpea (*Cice rarietinum* L.) is the second most important grain legume in the world next to common bean (*Phaseolus vulgaris* L.) and ranks third in world production, after common bean and field pea (*Pisum sativum* L.) [1]. The center of origin for chickpea are Turkey and Syria [2], but it was developed as a post-rainy season, spring-sown crop early in its history and expanded into sub-tropical countries, in contrast to its wild relatives, which have continued to grow as winter annuals in West and Central Asia [3]. Ethiopia is a secondary center of genetic diversity, and the crop is grown widely across the highlands and semi-arid regions of the country [4]. In terms of production and area coverage, it is currently grown on approximately 14.1 million ha worldwide, with an average annual production of 15.5 million tons [5].Desi and kabuli types are the two major types of chickpea grown in the world. Asia, Oceania, and Africa contribute 82%, 6%, and 5% of world production, respectively [6].In term of productivity in Ethiopia, chickpea is thirdamong legumes with production of 0.38 million tons and 0.08 million tons in desi and kabuli types, respectively [7].

Chickpea is one of the important pulse crops in Africa, particularly in Ethiopia, grown for human nutrition, provides 61% carbohydrates, 21% protein and 2.2% oil [8], income generation in local and foreign markets [9], improve soil fertility and acts as biocontrol of grassy weeds [10]. Even though the average global chickpea yield has improved during the past decades, productivity is still considered belowthe yield potential of the crop, which is 5 t ha-^1^ [11], while the average yield in Ethiopia is only 2 t ha-^1^ [7]. Several abiotic and biotic variables, such as frost, drought, water logging, weeds, diseases, insect pests, poor cultural practices, and low protection against pests, contribute to substantial yield gaps [12].

Among the factors responsible for low chickpea production, ascochyta blight (*Ascochyta rabiei)* is the most economically important disease in Ethiopia, when the chickpea growing season is cool and humid [13]. The disease causes significant quantity and quality yield losses up to 100% in most regions of the world and up to 41.3% in Ethiopia, depending on the climatic circumstances and susceptible cultivars [14]. As it isa seed-borne disease, seed can be the most important source of inoculum for long-distance dispersal [15].The disease affects all parts of chickpea plants, producing typical symptoms on the leaves, stems and pods, which rapidly turn into brown lesions with dark borders. As the disease progresses, small circular brown and black dots (pycnidia) develop in the center of these lesions and are arranged in concentric circles, the most important diagnostic feature [16]. In severe cases, the lesions enlarge and girdle the stem, and the entire plant dries up suddenly, leaving small patches of brown dead plants in the field [17].

The studies indicated that the disease distribution is becoming variable over seasons due to changes in climate conditions that favor disease development [18]. In addition, types of the variety grown, ecological area of the host, the high or low dose of fungicide applications and farming practices are sources of morphological, genetic, pathogenic and cultural variations within the pathogen population [19]. The intensity of disease across various agro-ecological zones varied in the amount and nature of distribution with different cultural, biological and physical environmental conditions [20]. Pathogen variation is one factor leading to plant disease epidemics and outbreaks, and it is one of the primary causes of plant disease management failures [19]. Currently, there are no adequate quantitative data on the distribution, prevalence and severity of ascochyta blight in the study areas rather than few studies on the disease prevalence. Moreover, no attempts have been made to correlate the biophysical factors with epidemics of thisdisease. Thus, the study of ascochyta blight disease epidemic could help to provide basic information on the disease distribution and its interaction with biophysical factors, important in designing effective disease management strategies in northwestern Ethiopia. Therefore, a field survey was conducted to assess the distribution, prevalence and intensity of ascochyta blight of chickpea and determine its association with agro-ecological factors in northwestern Ethiopia.

## Materials and methods

### Description of the survey areas

**Fig 1:**
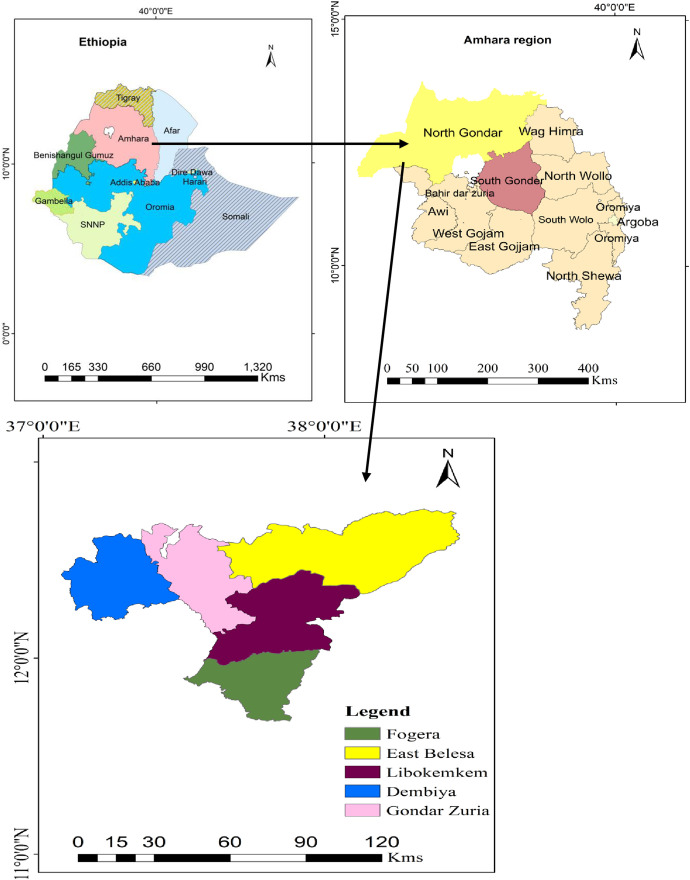
Map showing surveyed districts for ascochyta blight (*Ascochyta rabiei*) in northwestern Ethiopia, during the 2022 main cropping season. This figure is similar to map of Natural Earth but not identical.

**Fig 2:**
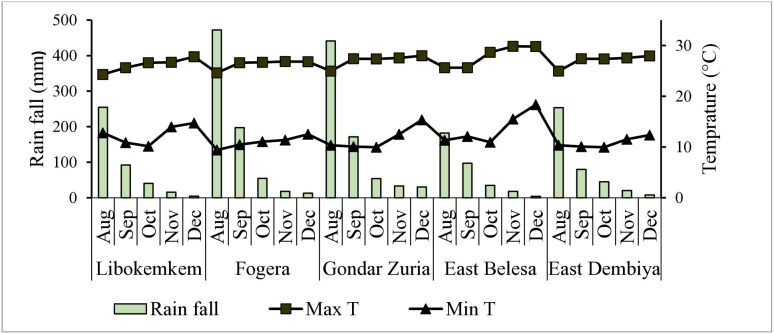
Monthly average rainfall (mm), maximum and minimum temperatures (^o^C) of surveyed districts in northwestern Ethiopia during the 2022 main cropping season. Max T = Maximum temperature, Min T = minimum temperature.

The field survey on the ascochyta blight epidemic was conducted in five potential chickpea-growing districts of northwestern Amhara National Regional State, Ethiopia (Fig 1).The districts were purposively selected based on their chickpea production potential. The districts included Fogera and Libokemkem in the South Gondar zone and Gondar Zuria, East Belesa, and East Dembiya districts in the Central Gondar zone. South Gondar is located at 11° 50’ 19“ N, longitude 38° 5’ 58” E, with an altitude range of 1786‒2921 metersabove sea level (m.a.s. l). The average annual rainfall of this zone is 1497 mm, with an average annual temperature of 17°C.Central Gondar zone is located at latitude 12° 39’ 60” N, longitude 37° 19’ 60” E, with an approximate altitude range of 1776‒2717 m. a. s. l. The annual mean maximum, mean, and mean minimum temperatures are 24.68, 18.09, and 11.5°C, respectively.The dominant soil types for both Zones are Vertisol and Nitisol [20,21].The major crops grown in the study area were *teff*, maize, grass pea, chickpea, barley, fenugreek, black cumin, white cumin, rice, sorghum, mungbean (Masho) and finger millet [22,23]. The rainfall, maximum and minimum temperatures of the surveyed districts during the disease assessment are presented in Fig 2.

### Sampling procedures and disease assessment

In five districts, four farmer associations were purposively selected based on their chickpea production status/potentialin consultation with the extension staff of the respective districts’ agriculture and rural development offices. In each farmers association, six chickpea farmer fields were selected randomly after a 3–5 km distance from the first field assessed. A total of 120 farmer fields were assessed.In each field, a quadrat (1 m × 1 m) was thrown by moving at 5 m intervals diagonally across each field from one end to the other in an “X” pattern. Chickpea fields were at two growth stages, such as pod setting andphysiologicalmaturity stage. During disease assessment in each field, total number of plants and plants showing ascochyta blight symptoms in a 1 m^2^ quadrat were counted. The whole plants within the quadrat were assessed for disease incidence, and 10 plants used for disease severity assessment from each quadrat at each field. Disease prevalence was determined by ratio of number of field showing chickpea disease to total number of fields assessed.

Ascochyta blight disease incidence was determined in each field on the basis of visual symptoms and by counting the number of symptomatic or infected plants from total plants in the quadrat and converting to percentage.The disease severity were assessed from randomly selected 10 plants in the quadrat that affected the area of a plant or plant organ and rated using severity scale of 1–9 [24] where,1 = no symptom; 2 = few, small, inconspicuous, up to 2 mm in size, occasionally present on some plant parts; 3 = few, scattered, larger, conspicuous, up to 5 mm but restricted in size; 4 = lesions obvious on some or all plant parts, may exceed 5 mm in size, defoliation initiated; 5 = lesions common, unrestricted in size, obvious on all plants/parts, defoliation and breaking and drying of branches slight to moderate; 6 = lesions as in 5, defoliation, broken, dry branches common, some plants killed; 7 = lesions as in 5, defoliation, broken, dry branches very common, up to 25% of the plants killed; 8 = symptoms as in 7, but up to 50% of the plants killed, and 9 = symptoms as in 7, but up to 100% of the plants killed were recorded for each field. The severity grades were converted into percent severity index (PSI) [25] for analysis. Disease incidence [20,26], prevalence [20,27] and severity were computed using the following formulas:


$$\text{Disease prevlance}=\frac{\text{Number of infected fields}}{\text{Total number of fields assessed}}x100$$

$$\text{Disease}\;\text{incidence}=\frac{\text{Number}\;\text{of}\;\text{diseased}\;\text{plants}\;\text{in}\;\text{the}\;\text{quadrat}}{\text{Total}\;\text{number}\;\text{of}\;\text{plants}\;\text{assessed}\;\text{in}\;\text{quadrat}}\times 100$$

$$\text{PSI}=\frac{\text{Sum}\;\text{of}\;\text{numerical}\;\text{ratings}}{\text{Number}\;\text{of}\;\text{plants}\;\text{scored}\;\times \;\text{maximum}\;\text{score}\;\text{on}\;\text{scale}}\times 100$$

In addition to disease data, planting date, previous crop history, crop variety, types of chickpea, seed source and their management options in the field were assessed byinterviewing the growers. The weed types, chickpea growth stages, cropping systems, soil types and weed management were recorded through observation, and weed and crop densities were noted by counting inside the quadrat. Geographic features like latitude, longitudeand altitude were recorded from all surveyed fieldsby a global positioning system.

### Data analysis

Descriptive analysis was performed to show the distribution, prevalence and association of ascochyta blight of chickpea disease intensity with biophysical factors across districts (Table 1). Class boundaries were selected for the disease incidence and severity into two distinct groups binomial qualitative data, i.e., ≤ 40% and >40% were chosen for disease incidence and ≤ 25% and > 25% for severity to form a binary dependent variable. Contingency table of disease incidence and severity and the independent variables were built to represent the bivariate distribution of fields according to data classifications (Table 1). The associations of ascochyta blight disease with agronomic practices and environmental factors were analyzed using a binary logistic regression model [28] using SAS procedure of GENMOD [29]. The importance of the independent variables was evaluated twice in terms of their effect on the incidence and severity. First, the association of all the independent variables was tested with theascochyta blight incidence and severity in a single-variable model. Second, the association of an independent variable with the disease incidence or severity was tested when entered first and last with all the other variables in the model.

**Table 1:** Categorizations of variables used in analysis for the distribution of ascochyta blight of chickpea epidemics in five districts of northwestern Ethiopia, during the 2022 main growing season.

Variable	Variable class	Number of Fields	Incidence (%)		Severity (%)	
			≤40	>40	≤ 25	>25
District	Libokemkem	24	15	9	15	9
	Fogera	24	8	16	8	16
	Gondar Zuria	24	9	15	9	15
	East Belesa	24	17	7	17	7
	East Dembiya	24	13	11	13	11
Cropping system^a^	Mixed cropping	42	9	33	9	33
	Sole cropping	78	53	25	53	25
Land preparation	1–2 plowing	52	17	35	17	35
	>2 plowing	68	45	23	45	23
Soil type	Vertisol	96	52	44	52	44
	Nitisol	24	10	14	10	14
Source of seed	Farmer saved	55	24	31	24	31
	Market	32	14	18	14	18
	Research center	33	24	9	24	9
Chickpea type	Desi	66	28	38	28	38
	Kabuli	54	34	20	34	20
Weed type	Broad leaf	58	20	38	20	38
	Grass	62	42	20	42	20
Previous crop^b^	Cereal	65	29	36	29	36
	Legume	55	33	22	33	22
Sowing date ^c^	August	54	22	32	22	32
	September	25	11	14	11	14
	October	41	29	12	29	12
Weed condition ^d^	Poor	53	17	36	17	36
	Good	67	45	22	45	22
Field size	≤ 0.5 ha	60	42	18	42	18
	> 0.5 ha	60	20	40	20	40
Growth stage	Pod setting	59	35	24	35	24
	Maturity	61	27	34	27	34
Attitude ^e^	≤1900	67	38	29	38	29
	>1900	53	24	29	24	29
Crop density ^f^	≤ 25	61	41	20	41	20
	>25	59	21	38	21	38

^a^Cropping system refers to planting only chickpea as sole and planting chickpea with other crops (safflower) as mixed.^b^Previously crop refers to crop that grew before chickpea planted in the same field. ^c^Sowing date refers to planting of chickpea late August starting from August 15–30, Sowing made in the beginning up to late September was considered as September and Sowing made in the beginning October considered as early October sowing. ^d^weeding condition was recorded as good and poor refers to weed‒free, and no weeding, respectively.^e^Altitude ≤ 1900 and >1900 m. a. s. l. are referred as low and highland areas, respectively. ^f^Crop population was determined in 1 m^2^ quadrat as highly populated (> 25) and less populated (≤ 25).

Lastly, those independent variables with significant association to the disease incidence or severity were added to a reduced multiple-variable model. The odds ratio was obtained by exponentiation of the parameter estimates for comparing the effect based on a reference point and interpreted as the relative risk. A complete analysis of the deviance reduction was calculated for each variable as it was added to the reduced multiple-variable model [28]. The GENMOD procedure fits a generalized linear model to the data with a maximum likelihood estimation of the parameter. The deviance, the logarithm of the ratio of two likelihoods, was used to compare the single and multiple variable models. The difference between the likelihood ratio tests (LRTs) was used to examine the importance of the variable and was tested against the Chi-square value [30].

## Results

### General characteristics of surveyed fields

The surveyed areas were in the range of altitudes 1722‒2150 m. a. s. l., out of 120, 67 (55.83%) of the fields were located ≤1900 m. a. s. l., while **53**fields (44.17%) were situatedabove 1900 m. a. s. l. During the field survey, about 55% of fields were planted with desi-type chickpea, and 45% of fields were planted with kabuli-type chickpea. All considered, districts grew Arerti, Haburu, and Dhera varieties of kabuli-type chickpea and Dalota, Eshete, and Geletuvarieties of desi-type chickpea at the time of diseaseassessment.About 45.83% of the fields were planted with farmer-saved chickpea seeds, and the rest were planted with seeds collected from local markets and research centers(Table 1). The majority of the farmersapplied weed management at the time of survey and almost all the weed types were grass (*Poa annua*, *Avena fatua*, *Digitaria sanguinalis*, *Setaria pumila*, *Eleusine indic a*and *Cynodon dactylon*) and broad leaf weeds (*Plantagolanceo*, *Datura stramonium*, *Solanum nigrum*, *Striga hermonthica* and *Galinsoga parviflora*). About 80%fields were Vertisol and 20% of fields were Nitisol soil type. The frequency of land preparation for chickpeas varied depending on agro-ecology; 68 (56.67%) fields were plowed more than 2 times, and 52 (43.3%) fields were plowed 1–2 times before sowing (Table 1).

**Fig 3:**
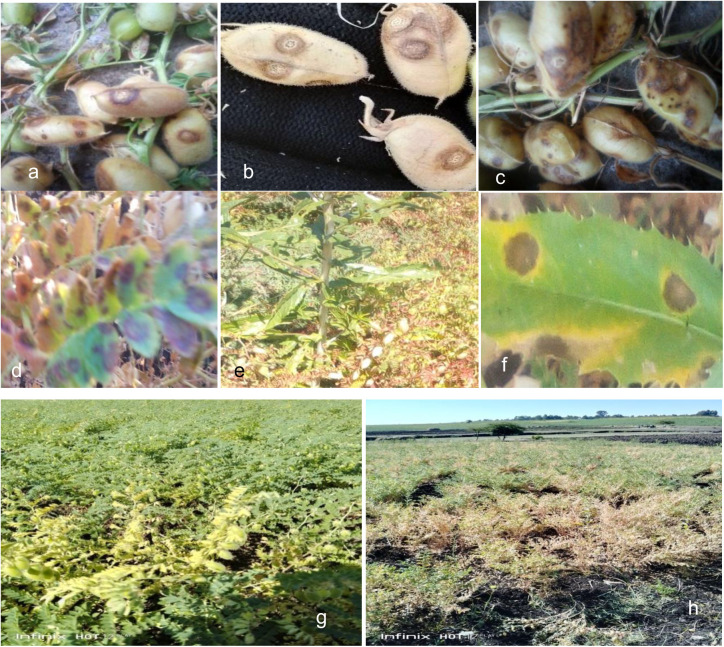
Ascochyta blight disease symptoms on chickpea plant and safflower in northwestern Ethiopia, during the 2022 surveyed season. Ascochyta blight symptoms on chickpea pods (a‒c), chickpea leaves (d), safflower leaves (e‒f), chickpea at podding (g) and maturity stage (h).

Chickpea populations varied between 12 and 42 plants per 1m^2^ depending on the types of cropping systems. Chickpea sowing was donein August, September and October months depending on the crop grown and rainfall distribution. Common crops cultivated in surveyed fields in the previous year were chickpea, grass pea, teff, sorghum, mung bean, rice, maize and finger millet. In this regard, 54.17% of chickpea fields were previously covered with cereals, and 45.83% of fields were covered by legumes.During disease assessment, chickpea fields were at podding (49.17%) and physiological maturity (50.83%) growth stages (Fig 3g, h).Most of farmers planted chickpea as sole (65%), while 35% of the fields were planted in mixed cropping system withsafflower (Table 1 and Fig 3e‒f).

### Prevalence, incidence and severity of ascochytablight

The field survey result indicated that ascochyta blight of chickpea was prevalent in all the surveyed areas. All assessed chickpea fields were infected by ascochyta blight and the prevalence was 100%.However, during field survey, different levels of disease incidence and severity of ascochyta blight of chickpea across districts were recorded. The highest (46.32%) disease incidence was obtained in Fogera, followed by Gondar Zuria (45.71%) and East Dembiya (40.19%) districts (Table 2 and Fig 3a–d). Conversely,lower disease incidence was assessed in East Belesa (31.26%) and Libokemkem (34.79%) districts. Similarly, the highest (32.90%) ascochyta blight severity was computed in Gondar Zuria, followed by Fogera (29.43%) district, while the lowest disease severity was recorded in Libokemkem (21.29%) and East Belesa (22.90%) districts (Table 2).

**Table 2:** Incidence and severity (mean ± SE) of ascochyta blight of chickpea for different independent variables during the 2022main growing season, northwestern Ethiopia.

Variable	Variable class	Incidence (%)	SE	Severity (%)	SE
District	Libokemkem	34.79	2.23	21.29	1.65
	Fogera	46.32	3.27	29.43	2.21
	Gondar Zuria	45.71	2.26	32.90	2.80
	East Belesa	31.26	2.82	22.90	1.52
	East Dembiya	40.19	3.63	24.52	2.13
Cropping system^a^	Mixed cropping	50.41	2.18	30.86	1.86
	Sole cropping	33.87	1.39	23.70	1.09
Land preparation	≤2 times plowing	42.50	2.05	28.74	1.60
	>2 times plowing	37.48	1.84	24.27	1.15
Soil type	Vertisol	39.92	1.58	27.98	1.84
	Nitisol	38.58	2.86	25.76	2.06
Seed source	Farmer saved	42.75	2.02	27.24	1.43
	Market	40.90	2.87	27.00	1.67
	Research center	33.29	2.21	23.71	2.26
Chickpea type	Desi	41.45	1.88	27.52	1.31
	Kabuli	37.46	2.02	24.59	1.55
Weed type	Broad leaf	43.54	1.80	27.75	1.45
	Grass	36.02	1.99	24.76	1.39
Previous crop^b^	Cereal	37.23	1.94	24.26	1.39
	Legume	41.70	1.92	27.85	1.43
Sowing date ^c^	August	43.36	1.84	27.62	1.48
	September	41.88	3.69	27.60	2.64
	October	33.41	2.07	23.49	1.49
Weeding condition^d^	Poor	44.88	1.71	28.31	1.38
	Good	35.52	1.94	24.54	1.42
Field size	≤0.5 ha	34.30	1.86	24.64	1.53
	>0.5 ha	45.00	1.81	27.77	1.29
Growth stage	Podding	37.20	2.06	25.15	1.48
	Maturity	42.02	1.82	27.23	1.37
Altitude (m a. s. l.)^e^	≤ 1900	37.48	1.73	25.27	1.35
	>1900	42.41	2.20	27.39	1.51
Cropdensity(m^‒2^)^f^	≤ 25	35.71	1.89	23.65	1.37
	>25	43.73	1.89	28.85	1.40

^a^Cropping system refers to planting only chickpea as sole and planting chickpea with other crops (saff flower) as mixed. ^b^Previously crop refers to crop that grew before chickpea planted in the same field. ^c^Sowing date refers to planting of chickpea early August up to late August consider as August planting, early September up to late September as September planting and the beginning of October up to late October also as October sowing. ^d^Weeding condition was recorded as good and poor refers to weed‒free, and no weeding, respectively. ^e^Altitude ≤1900 and >1900 m. a. s. l. are referred as low and high altitude areas, respectively. ^f^Crop population was determined in 1 m^2^ quadrat as highly populated (> 25) and less populated (≤ 25).

During the field survey, the highest disease incidence (50.41%) and severity (30.86%) were assessed from mixed cropping compared to sole cropping system (Table 2). Chickpea fields’ plowed ≤ 2times exhibited higher disease incidence (42.50%) and severity (28.74%) than field plowed more than two times (Table 2). Relatively, the highest mean ascochyta blight incidence (39.92%) and severity (27.98%) were recorded from the chickpea grown in Vertisol compared to chickpea cultivated in Nitisol (Table 2). Among chickpea type, the highest disease incidence (41.45%) and severity (27.52%) were recorded fromthe fields planted with desi type than kabuli type. On the other hand, high incidence (43.54%) and severity (27.75%) were scored in fields infested with broad leaf weed types compared to fields with grass weeds (Table 2). Regarding weed management, higher disease incidence (44.88%) and severity (28.31%) were recorded in field with poor weed management than in well-managed fields (Table 2).

Fields that had previously been planted with legumes had a greater incidence (41.70%) and severity (27.85%) than cultivated chickpeas after cereal crops (Table 2).Planting chickpea in August and September exhibited higher disease incidence andseveritythan sowing of chickpea in October (Table 2). In this survey, the highest disease incidence (42.02%) and severity (27.23%) were assessed atphysiological maturity stage than podding stage (Table 2). Comparatively, fields at high altitudes (> 1900 m a. s. l.) exhibited higher disease incidence (42.41%) and severity (27.39%) compared to fields at low altitudes (≤ 1900 m a. s. l.).Crop population of > 25 chickpea plants per m^2^ had higher disease incidence (43.73%) and severity (28.85%) than sparsely populated (≤ 25 chickpea plants per m^2^) chickpea fields (Table 2).

### Association of ascochyta blight Disease Intensity with biophysical factors

The associations of all agro-ecological factors with the disease intensity are presented in Table 3. The biophysical factors, including land preparation and cropping system, showed highly significant (P < 0.0001) associations with ascochyta blight intensity in the logistic model as a single variable, and they also maintained their associations with both incidence and severity when included into the reduced variable model (Table 3). District showed significant (P < 0.03) relation with disease intensity when entered first into the model and highly significant (<0.005) association when added last into model along with other variables. Besides, soil type and weed type were showed significant association with the disease intensity in the model when entered first and last into the model.Chickpea typewas significantly correlated with both disease incidence and severity when tested as first variable into the model and lost their significance when entered last into the reduced model (Table 3).

**Table 3:** Logistic regression model of ascochyta blight disease of chickpea incidence and severity (PSI), and likelihood ratio test for independent variables in northwestern Ethiopia during the 2022 main cropping season.

Independent Variable	df	Ascochyta blight incidence, LRT^a^				Ascochyta blight PSI, LRT			
		Type 1 analysis (VEF)		Type 3 analysis (VEL)		Type 1 analysis (VEF)		Type 3 analysis (VEL)	
		DR	Pr > χ²	DR	Pr > χ²	DR	Pr > χ²	DR	Pr > χ²
District	4	10.08	0.03	14.65	0.005	10.08	0.03	14.65	0.005
Cropping system	1	23.01	<0.0001	7.65	0.005	23.01	<0.0001	7.65	0.005
Land preparation	1	14.33	0.0001	14.08	0.0002	14.33	0.0002	14.08	0.0001
Soil type	1	5.05	0.03	6.26	0.01	5.05	0.04	6.26	0.01
Source of seed	2	1.85	0.40	2.35	0.31	1.85	0.40	2.35	0.31
Chickpea type	1	5.31	0.02	2.65	0.10	5.31	0.03	2.65	0.11
Weed type	1	4.58	0.04	4.51	0.03	4.58	0.03	4.51	0.02
Previous crop	1	1.74	0.19	1.15	0.28	1.74	0.19	1.15	0.28
Sowing date	2	0.31	0.86	0.33	0.85	0.31	0.86	0.33	0.85
Weed condition	1	1.94	0.16	1.74	0.19	1.94	0.16	1.74	0.19
Field size	1	0.81	0.37	0.78	0.38	0.81	0.37	0.78	0.38
Growth stage	1	0.24	0.62	0.42	0.52	0.24	0.62	0.42	0.52
Altitude	1	0.26	0.61	0.22	0.64	0.26	0.61	0.22	0.64
Plant density	1	0.17	0.68	0.17	0.68	0.17	0.68	0.17	0.68

^a^LRT = likelihood ratio test, VEF = Variable entered first in the mode, VEL = Variable entered last in the model, DR = deviance reduction, Pr = Probability of a χ2 value exceeding the deviance reduction, χ2 = Chi-square, df = Degrees of freedom, PSI = Percent severity index.

However, other factors such as, previous crop history, sowing date, weed condition, field size, growth stage, altitude and plant density were not significantly associated with disease incidence and severity when tested first or last into model along with other variables(Table 3. The non-significant association of factors with disease intensity implies that the variables have less or no influence on chickpea ascochyta blight disease epidemic development. Moreover, some of these variables were less important to decrease or increase the chickpea ascochyta blight disease epidemic than other variables.

All significantly associated independent variables were tested in the reduced multiple-variable model and their analysis of deviancehighlighted importance of each variable and respective variable class toascochyta blight intensity. The results of the analysis of deviation for each variable and variable class to incidence and severity, parameter estimates, standard error, and odds ratio are given in Tables 4 and 5.

**Table 4:** Analysis of deviance, natural logarithms of odd ratio and standard error of ascochyta blight of chickpea incidence, and likelihood ratio test on independent variables during the 2022 main growing season, northwestern Ethiopia.

Added variable ^a^	Residual deviance^b^	df	Ascochyta blight incidence, LRT^c^		Variable class	Estimate^d^	SE	Odds Ratio
			DR	Pr > χ²				
Intercept	166.22					−0.88	0.85	0.41
District	156.14	4	10.08	0.03	Libokemkem	−2.29	0.86	0.10
					Fogera	0.79	0.80	2.20
					Gondar Zuria	0.98	0.84	2.67
					East Belesa	−0.71	0.81	0.49
					East Dembiya	0^*^		1
Cropping system	133.13	1	23.01	<.0001	Mixed cropping	2.14	0.66	8.50
					Sole cropping	0^*^		1
Land preparation	118.79	1	14.33	0.0001	≤2 plowing	2.41	0.65	11.11
					>2 plowing	0^*^		1
Soil type	113.75	1	5.05	0.02	Nitisol	−2.00	0.80	0.13
					Vertisol	0^*^		1
Source of seed	111.90	2	1.85	0.40	Farmer saved	−0.39	0.75	0.68
					Market	0.51	0.75	1.67
					Research center	0^*^		1
Chickpea type	106.59	1	5.31	0.02	Desi	1.12	0.54	3.06
					Kabuli	0^*^		1
Weed type	102.00	1	4.58	0.04	Broad leaf	1.39	0.63	4.01
					Grass	0^*^		1
Previous crop	100.26	1	1.74	0.19	Cereal	−0.76	0.60	0.47
					Legume	0^*^		1

^a^Variables added into the model in order of presentation in Table. ^b^Unexplained variations after fitting the model. ^c^LRT = Likelihood ratio test. DR = Deviance reduction; Pr = Probability of a χ2 value exceeding the deviance reduction. χ2 = Chi‒square. ^d^Estimates from the model with all independent variables added.

**Table 5:** Analysis of deviance, natural logarithms of odds ratio, and standard error of added variables in a reduced model of severity ascochyta blight of chickpea in northwestern Ethiopia during the 2022cropping season.

Added variable^a^	Residual deviance^b^	df	Ascochyta blight severity, LRT^c^		Variable class	Estimate ^d^	SE	Odds Ratio
			DR	Pr > χ²				
Intercept	166.22				Intercept	−0.79	0.89	0.45
District	156.14	4	10.08	0.02	Libokemkem	−2.36	0.87	0.09
					Fogera	0.73	0.81	2.08
					Gondar Zuria	1.00	0.84	2.71
					East Belesa	−0.79	0.83	0.45
					East Dembiya	0^*^		1
Cropping system	133.13	1	23.01	<.0001	Mixed cropping	2.19	0.67	8.93
					Sole cropping	0^*^		1
Land preparation	118.79	1	14.33	0.0002	≤2 plowing	2.44	0.66	11.42
					>2 plowing	0^*^		1
Soil type	113.75	1	5.05	0.04	Nitisol	−2.01	0.83	0.13
					Vertisol	0^*^		1
Seed source	111.90	2	1.85	0.40	Farmer saved	−0.16	0.85	0.86
					Market	0.64	0.79	1.90
					Research center	0^*^		1
Chickpea type	106.59	1	5.31	0.03	Desi	1.15	0.54	3.15
					Kabuli	0^*^		1
Weed type	102.00	1	4.58	0.03	Broad leaf	1.48	0.66	4.41
					Grass	0^*^		1
Previous crop	100.26	1	1.74	0.19	Cereal	−0.80	0.62	0.45
					Legume	0^*^		1

^a^Variables added into the model in order of presentation in Table. ^b^Unexplained variations after fitting the model. ^c^LRT = Likelihood ratio test. DR = Deviance reduction. Pr = Probability of a χ2 value exceeding the deviance reduction. χ2 = Chi‒square. ^d^Estimates from the model with all independent variables added.

Logistic regression analyses indicated that Fogeraand Gondar Zuria districts, mixed cropping system, ≤ 2 times ploughing and broad leaf weed type were associated with the ascochyta blight intensity and had significant contributions to the development of epidemics (Tables 4 and 5). However, variable classes such as, Libokemkem and East Belesa districts, sole cropping system and more than 2 times ploughing revealed significant association with low ascochyta blight incidence (≤ 40%) and severity (≤ 25%). The probability of the highest incidence (> 40%) relationships with Gondar Zuria and Fogera districts were 2.20and 2.67times greater than East Dembiya district, respectively. Likewise, the probability of highest (> 25%) disease severity associations with Gondar Zuria and Fogera were 2.71 and 2.08 times greater than East Dembiya, respectively. Growing of chickpea at Libokemkem and East Belesa districts was about 91% and 55% less risk than East Dembiya district. The survey result indicated that growing of chickpea in mixed croppingwas highly associated with the highest disease incidence (> 40%) and severity (> 25%) by8.5 and 8.93 times greaterthan growing of chickpea in sole cropping system, respectively.

The probability of highest disease incidence (>40%) and severity (> 25%) in chickpea fields plowing with less than or equal to two times was 11 times and nearly 12 times higher than chickpea field plow more than two times, respectively.Regarding soil types, the epidemic of ascochyta blight was 87% less risk on growing chickpea in Nitisol than cultivating the crop in Vertisol (Tables 4 and 5). Chickpea fields planted with desi type was highly associated with high levels of disease incidence and severity compared to fields planted with kabuli chickpea type. Weed type was also important factors for ascochyta blight disease development. In this survey, fields infested with broadleaf weed type were significantly associated with highascochyta blight disease incidence and severity, which were nearly 4 and 4.5 times higher probability of occurrence of ascochyta blight incidence and severity than chickpea fields with grassy weeds, respectively (Tables 4 and 5).

## Discussion

The disease was widely distributed and prevalent across geographical locations. All assessed chickpea fields were infected by ascochyta blight and the prevalence was 100%.The distribution of the disease in all surveyed locations may be due to the presence of variable pathogen races in the surveyed areas, which enable the pathogen to infect the host widely regardless of agro-ecological differences. Previous studies [31] reported that ascochyta blight is present in almost all chickpea-cultivating regions across the world and is deemed to be the most devastating biotic factor, causing significant loss of yield and degradation of seed quality.Moreover, the wide distribution of ascochyta blight disease may be due to the farming practices adopted bysmallholder farmers in the study areas.The reports [32] available in support of the present findings, that the use of poor-quality farmer-saved seed, lack of crop rotation due to land scarcity, and poor management practices wereexacerbatedby the environmental conditions forthe disease development.

In the current study, the higher disease incidence and severity of ascochyta blight in poorly managed chickpea fields could be attributed to high weed infestation,which may reduce crop vigor through space and nutrient competition, hence promoting disease development.In different host-pathogen systems, competition for space and soil nutrients in highly weed-infested fields has been found to render crops susceptible to both foliar and soil-borne pathogens [33]. Furthermore, the weeds may harbor pathogens from the preceding cropping season acting as alternative hosts or as symptomless carriers. Hence, may serve as a source of inoculum [34].Regarding the previous crop, cultivated chickpea following legume had relatively higher ascochyta blight incidence and severity than chickpea planted after cereal crops (Table 2). This could result in the buildup of more inoculum due to growing host crops and legumes year after year, accumulating ascospores [35]. Hence, [36] it has been proposedthat removing preceding hosts is the best option tointerrupt plant pathogen’s life cycle.

Planting chickpea in August and September had higher ascochyta blight incidence and severity than sowing of chickpea in October (Table 2). This may be due to the presence of high rainfall and humidity in August and September (Fig 2),favoring disease establishment by providing more time and suitable conditions for epidemic development. Moreover, late planting delayed the time to disease onset [37], implying that favorable conditions did not coincide and interact at the appropriate time or for a sufficient duration. In the present survey, disease incidence and severity varied between chickpea growth stages. In this regard, the highest ascochyta blight incidence and severity were assessed at physiological maturity stage than podding stage (Table 2). Thismay be associated with the existence of active cells in vegetative and young growth stage that defend plants from pathogen attack. In this context, pathogens can easily penetrate and develop in the crop due to the loss of crop rigidity and phenolic compounds during crop maturity [38,39] noted that crop susceptibility to ascochyta blight increased as the crop matured, which could be attributed to higher expression of resistance genes in post-seedling to vegetative growth stages than at maturity growth stages and decreased phytoalexin concentrations in old chickpea tissue [15].

Regarding the chickpea population, densely populated chickpea fields showed higher disease incidence and severity than sparsely populated chickpea fields.This might be due to a dense plant population reducing air circulation and resulting in a more humid environment that promotes blight disease development. A very close availability of host plants intercepts the inoculum and reduces the chance of it being lost to the soil [40]. Furthermore, dense crop populations increase competition for resources, potentially weakening host plants and making them more susceptible to pathogens, hence increasing disease epidemics and reducing crop yield [41]. In the present study, Gondar Zuria and Fogera districts have a greaterrisk of experiencing the highdisease incidence and severity than other districts. Variations in the intensity of ascochyta blight have been recorded in surveyed districts in Central Ethiopia [40] and in the central Alberta districts of Canada [42]. For instance, 0‒45.6% of disease incidence in Ethiopia [43] and 18‒100% of severity in Syria [44] were estimated in the surveyed area.This variation could be attributed to agro-ecological difference among districts. Moreover, continuous cultivation of chickpeas over the years, provide an inoculum reservoir for disease establishment and epidemics, and differences in farmersmanagement practice. Furthermore, as the fields in Gondar Zuria and Fogera districts were flat and the majority of soil types were vertisol, high soil moisture levels may have been preserved, creating favorable conditions for ascochyta blight epidemics.

The current survey results indicated that growing chickpea in mixed cropping was highly associated with the highest disease intensity compared to the chickpea grownin sole cropping system. The presence of safflower with chickpea in mixed cropping system may increase the relative humidity owing to its branching and taller height than the host crop, providing shade effect and more time for leaf wetness, favoring disease establishment and development (Fig 3e). Mixed cropping systems also hinder air circulation inside the crop fieldby increasing relative humidity, which creates favorableconditions for disease development [45]. Moreover, increasing incidence of disease epidemics in mixed cropping systems may be due to the occurrence of ascochyta blight symptoms on safflower thatacts as an alternative host for the disease (Table 2 and Fig 3e). In the present study, the chickpea fields plowed more than two times were associated with less disease intensity. This could be due to the exposure of the initial inoculum to solar energy,leading to the reduction inthe amount of inoculum in the soil, and the decomposition of the alternative host, leadingto early disease establishment.According to [46], frequent plowing could reduce the survival of pathogen populationsin the soil via mechanical disturbance by rapid breakdown of infected crop residues. On the other hand, previous studies in the United States [47] reported that less frequent plowing practices maintain most crop residues on the soil surface that may increase ascochyta blight epidemics of chickpeas.

Regarding soil types, the epidemic of ascochyta blight is more highly associated with chickpeas grown in Nitisol than cultivating the crop in Vertisol(Tables 4 and 5).This could be due to the presence of small soil micro pores that conserve soil moisture and reduce soil temperatures, creating suitable conditions for the pathogen’spseudothecia formation. This association could be attributed to the higher moisture retention capacity of dark soil (Vertisol) compared to light soil, which favorsthe development of ascochyta blight pseudothecia [48]. Chickpea fields planted with desi type were highly associated with higher levels of disease incidence and severity compared to fields planted with kabuli chickpea type. The highest disease epidemic association with the desi type of chickpea might be related to the pathogen adaptation to the crop resistance mechanism, presence of a virulent pathogen population, and the deployment of the qualitative type of resistance in the desi type.A previous study [49] reported lower ascochyta blight disease incidence and severity in kabuli type (compound leaves) than in desi type (unifoliate).Weed type also played an essential role in the development of ascochyta blight disease. In this survey, fields infested with broadleaf weedshad a much higher incidence and severity of ascochyta blight. This could be due to the large canopy size in broadleaf weeds, increasingrelative humidity, which is suitable for disease development.A dense canopy reduces airflow, resulting in a more humid microclimate conducive to blight development [50].

## Conclusion

A survey of 120 fields for ascochyta blight of chickpea (*Ascochyta rabiei*) during the 2022 main cropping season revealed that it was the most prevalent and widely distributed disease in all chickpea-growing fields in the study areas and varied among districts. The ascochyta blight incidence and severity varied among districts and farming practices, along with biological and environmental factors.The logistic regression analysis confirmed that independent variables, such as districts (Gondar Zuria and Fogera), mixed cropping system, less than two times plowing, Vertisol soil type, desi chickpea type and broadleaf weed had high probability of association with ascochyta blight epidemics. Conversely, ascochyta blight disease intensity were lower in Libokemkem and East Belesa districts, sole cropping system, more than two times plowing, Nitisol and kabuli chickpea type. Thus, proper weeding practices, plowing (>2 times), and planting kabuli chickpea types in Nitisol could be considered as alternate management options to reduce the epidemic of ascochyta blight. However, further comprehensive surveys in two seasons and different agro-ecological zones involving legumes, non-legume crops, and weed species are required to generate a better conclusion about the association of relevant biophysical factors with disease intensity. In addition, molecular research is important to understand the genetic diversity of *Ascochyta rabiei* isolates in the studied areas.
